# Unique and Conserved Endoplasmic Reticulum Stress Responses in Neuroendocrine Cells

**DOI:** 10.3390/cells14191529

**Published:** 2025-09-30

**Authors:** Karina Rodrigues-dos-Santos, Gitanjali Roy, Anna Geisinger, Sahiti Somalraju, Travis S. Johnson, Michael A. Kalwat

**Affiliations:** 1 Indiana Biosciences Research Institute, Indianapolis, IN 46202, USA; 2 Department of Biostatistics, Indiana University School of Medicine, Indianapolis, IN 46202, USA; 3 Center for Diabetes and Metabolic Diseases, Indiana University School of Medicine, Indianapolis, IN 46202, USA

**Keywords:** endocrinology, ER stress, hormone secretion, computational biology

## Abstract

**Highlights:**

**What are the main findings?**

- ER stress induces core unfolded protein response genes across neuroendocrine cell types, as well as unique transcriptional programs.
- Comparative transcriptomics reveals concordant and discordant responses to ER stress, highlighting potential roles of novel gene candidates.

**What are the implications of the main findings?**

- Discordant gene expression may explain differences in susceptibility to ER stress-induced dysfunction and death.
- An interactive web tool enables custom end-user analysis of neuroendocrine cell ER stress responses.

**Abstract:**

Endocrine cells are dedicated to the production and processing of hormones, from peptides to small molecules, to regulate key physiological processes, including glucose homeostasis and metabolism. Because of this relatively high productivity, endocrine cells must handle a variety of stresses from oxidative stress to the unfolded protein response of the endoplasmic reticulum (UPR^ER^). While much is known about the major pathways regulating the UPR^ER^, the roles of endocrine cell type-specific, context-dependent, and time-dependent transcriptional changes are not well explored. To identify unique and shared responses to the UPR^ER^ across a subset of endocrine cell types, we tested representative lines for β-cells (insulin), α-cells (glucagon), δ-cells (somatostatin), X/A-cells (ghrelin), L-cells (glucagon-like peptide 1 (GLP1)), and thyrotropes (thyroid hormone and thyroglobulin). We exposed each cell type to the canonical ER stressor thapsigargin for 6 and 24 h, or vehicle for 24 h, and performed mRNA sequencing. Analysis of the data showed all lines responded to thapsigargin. Comparisons of differentially expressed genes between each line revealed both shared and unique transcriptional signatures. These data represent a valuable mineable set of candidate genes that may have cell type-specific functions during the UPR^ER^ and have the potential to lead to a new understanding of how different endocrine cells mitigate or succumb to ER stress.

## 1. Introduction

The unfolded protein response of the endoplasmic reticulum (UPR^ER^) is conserved across eukaryota and is engaged when the burden of secretory protein production exceeds capacity, inducing a gene program to mitigate stress or, if mitigation is not achieved, induce apoptosis [1,2]. The UPR^ER^ has been an attractive source of pharmacological targets for diseases, including diabetes and cancer [3]. Dedicated secretory cell types, like neuroendocrine cells, have a unique reliance on these stress pathways and represent useful models for studying the mechanisms underlying ER stress and the subsequent activation of UPR^ER^ [4,5,6]. The UPR^ER^ involves activation of three canonical signaling pathways: PERK-eIF2α-ATF4; IRE1α-XBP1s; and ATF6 [7]. The UPR^ER^ is a double-edged sword in certain endocrine cells, like pancreatic islet β-cells. β-cells require the UPR^ER^ for normal function, but in diseases like type 1 diabetes (T1D) and type 2 diabetes (T2D), chronic engagement of the UPR^ER^ may exacerbate β-cell failure. The relative cell-type-specific signatures of UPR^ER^ are not well defined across disparate types of endocrine cells. Characterizing these unique signature genes may enable the identification of novel cell-type-specific biomarkers and targets, as well as new insights into the underlying biology, thereby improving the understanding of the UPR^ER^ in general.

In this study, we have generated a transcriptomic resource comparing the responses of a panel of dedicated secretory cell types to the canonical ER stressor thapsigargin (Tg). We performed computational analyses to cross-compare all cell types and identify unique and shared transcriptomic responses. We have focused here on the unique UPR^ER^ responses of β-cells and α-cells, given the preponderance of data indicating α-cells have a distinct response to stress, which may contribute to their survival in T1D [8]. However, we expect that these data will be useful across multiple fields and have provided an interactive web tool to facilitate UPR^ER^ comparisons.

## 2. Materials and Methods

### 2.1. Compounds

Thapsigargin was obtained from Adipogen (San Diego, CA, USA) (AGCN20003M001). All other chemicals were from reputable sources, including Sigma-Aldrich (St. Loius, MO, USA) and Fisher Scientific (Waltham, MA, USA).

### 2.2. Cell Culture, Treatments, and RNA Isolation

MIN6 β-cells (RRID: CVCL_0431) were cultured in high-glucose DMEM containing 10% fetal bovine serum (FBS), 50 µM β-mercaptoethanol, 4 mM L-glutamine, 1 mM pyruvate, 100 U/mL penicillin, and 100 μg/mL streptomycin. The mouse intestinal L cell line GLUTag (RRID: CVCL_J406) was provided by G.G. Holz (Upstate Medical University) and was cultured in low-glucose DMEM containing 10% FBS, 4 mM L-glutamine, 1 mM pyruvate, 100 U/mL penicillin, and 100 μg/mL streptomycin. Human somatostatinoma QGP-1 (RRID: CVCL_3143) cells [9] were provided by Dawn E. Quelle (University of Iowa) and were cultured in RPMI-1640 with 10% FBS, 1 mM pyruvate, 4 mM L-glutamine, 100 U/mL penicillin, and 100 μg/mL streptomycin. The mouse ghrelinoma cell line MGN3 (RRID: CVCL_C4PL) [10] was provided by Hiroshi Iwakura (Wakayama Medical University, Japan) and was cultured in high-glucose DMEM supplemented with 10% FBS and containing 4 mM L-glutamine, 100 U/mL penicillin, and 100 µg/mL streptomycin. Tissue culture plates were pre-coated with diluted Matrigel (1:20) for 1 h at 37 °C prior to seeding. The rat thyroid cell line PCCL3 (RRID: CVCL_6712) [4] was provided by Peter Arvan (University of Michigan) was cultured in Coons F-12 supplemented with 1 mIU/mL thyrotropin, 1 µg/mL insulin, 5 µg/mL apo-transferrin, 1 nM hydrocortisone, 5% FBS, 100 U/mL penicillin, and 100 μg/mL streptomycin. The mouse pancreatic α-cell line αTC1 clone 6 (αTC1-6) (RRID: CVCL_B036) [11] was provided by Michael Roth (UT Southwestern). αTC1-6 cells were cultured in 15 mM glucose DMEM supplemented with 15 mM HEPES, 0.1 mM non-essential amino acids, 10% FBS, 0.03% BSA, 100 U/mL penicillin, and 100 μg/mL streptomycin. GLUTag, QGP-1, PCCL3, aTC1, and MGN3 and MIN6 cells were plated in 6-well dishes and exposed to DMSO 0.1% or thapsigargin 100 nM (GLUTag, QGP-1, and MGN3 and MIN6) or 500 nM (PCCL3 and αTC1-6) for 6 and 24 h before harvesting for RNA purification, downstream gene expression analysis, and RNAseq. A higher dose of thapsigargin was used for PCCL3 and αTC1-6 cells based, in part, on the observed cellular morphology after 24 h of treatment and on published data showing that effects of 100 nM thapsigargin require 48 h exposure [12] to substantially impact PCCL3, and that α-cells are known to be resistant to ER stress conditions [8]. For cell viability/death assays, the MultiTox-Fluor kit from Promega (Madison, WI) was used following the manufacturer’s instructions. Briefly, MIN6 and αTC1 cells were seeded into 96-well black-walled clear-bottom tissue culture dishes at 10^5^ cells/well. Cells were cultured until reaching 80–90% confluency before treatment with DMSO 0.1% or thapsigargin for 24 h. On the day of the assay, cell-permeant GF-AFC substrate (live cell readout) and cell-impermeant bis-AAF-R110 substrate (dead cell readout) reagents were diluted into assay kit buffer as a 2X working solution. Then, 100 μL of 2X working solution was added to each well of cells in 100 µL of medium and incubated for 30 min at 37 °C. Plates were read on a BioTek Synergy H1M2 plate reader (400Ex/505Em for live cells; 485Ex/520Em for dead cells).

### 2.3. Human Islet Culture and Treatment

Cadaveric human islets were obtained through the Integrated Islet Distribution Program (IIDP) and Prodo Labs. Islets were isolated by the affiliated islet isolation center and cultured in PIM medium (PIM-R001GMP, Prodo Labs (Aliso Viejo, CA, USA)) supplemented with glutamine/glutathione (PIM-G001GMP, Prodo Labs), 5% human AB serum (100512, Gemini Bio Products (West Sacramento, CA, USA)), and ciprofloxacin (61-277RG, Cellgro, Inc. (Lincoln, NE, USA)) at 37 °C and 5% CO_2_ until they were shipped at 4 °C overnight. The human islets were cultured upon receipt in complete CMRL-1066 (containing 1 g/L (5.5 mM) glucose, 10% FBS, 100 U/mL penicillin, 100 μg/mL streptomycin, and 292 µg/mL L-glutamine). Human islet information and metadata from three different donors are provided in Table S1. The human islet experiments were performed using islets from each donor independently. For drug treatments, ~50 human islets were hand-picked under a dissection microscope, transferred into low-binding 1.5 mL tubes, and cultured in 500 µL of complete CMRL-1066 medium containing DMSO (0.1%) for 24 h or thapsigargin (1 μM) for 6 and 24 h. The dose of thapsigargin was chosen based on prior work in human islets and the human β-cell line EndoC-βH1 [13,14,15,16].

### 2.4. RNA Isolation and RT-qPCR Validation

ER stress was induced in the cell lines and human islets using thapsigargin as described for 6 and 24 h. After the indicated treatments in cell lines, the medium was removed from the cells, and lysis buffer (with β-mercaptoethanol) was added to the cells. The cells were scraped, and the lysates were transferred to 1.5 mL tubes on ice and then transferred to −80 °C for storage; RNA was later isolated using the Aurum Total RNA Mini Kit (Bio-Rad (Hercules, CA, USA)). For the human islets, the islets were harvested and RNA isolated using the Quick-RNA Microprep (Zymo (Irvine, CA, USA)). RNA concentration was measured using a Nanodrop spectrophotometer and verified to have A260/280 ratios > 2.0. RNA (1 μg for cell lines, 500 ng for islets). RNA was converted into complementary DNA (cDNA) using the iScript cDNA synthesis kit (Bio-Rad) following the manufacturer’s instructions. cDNAs were diluted 10-fold, and 1 µL was used per qPCR reaction to confirm the induction of ER stress responses. One microliter of diluted cDNA was used in 10 μL quantitative polymerase chain reactions using 2X SYBR Bio-Rad master mix and 250 nM of each primer. Reactions were run in a 384-well format on QuantStudio 5 (Thermo, Waltham, MA, USA). qPCR data were analyzed using the QuantStudio software with *18S* RNA as the reference gene for cell lines and *ACTB* and *VAPA* as reference genes for human islets [17]. Relative expression was calculated by the 2^−ΔΔCt^ method. All primer sequences are provided in Table S2.

### 2.5. Transcriptomics

For MIN6 β-cells, samples were sequenced previously according to the same protocol below, and the read count data for DMSO 24 h, Tg 6 h, and Tg 24 h treatments were obtained from GSE194200 [18]. For αTC1-6, QGP-1, MGN3, PCCL3, and GLUTag cells, 300 ng of RNA was submitted to the Indiana University Center for Medical Genomics for mRNA-seq as described previously [18]. Total RNA samples were first evaluated for their quantity and quality using an Agilent Bioanalyzer 2100 (Agilent (Santa Clara, CA, USA)). All samples were of good quality with RIN (RNA Integrity Number) ≥ 9. One hundred nanograms of total RNA was used for library preparation with the KAPA mRNA Hyperprep Kit (KK8581) on Biomek following the manufacturer’s protocol. Each uniquely dual-indexed library was quantified and quality-assessed by Qubit (Fisher Scientific (Waltham, MA, USA)) and TapeStation (Agilent (Santa Clara, CA, USA)), and multiple libraries were pooled in equal molarity. The pooled libraries were sequenced with 2X 100 bp paired-end configuration on an Illumina NovaSeq 6000 sequencer (Illumina, (San Diego, CA, USA)) with the v1.5 reagent kit. Samples had an average read depth of ~52.7 million reads/sample. The sequencing reads were first quality-checked using FastQC (v.0.11.5, Babraham Bioinformatics, Cambridge, UK) for quality control. The sequence data were then mapped to either the mouse reference genome mm10, the human reference genome hg38, or the rat reference genome rn6 using the RNA-seq aligner STAR (v.2.710a) [19] with the following parameter: “--outSAMmapqUnique 60”. To evaluate the quality of the RNA-seq data, the number of reads that fell into different annotated regions (exonic, intronic, splicing junction, intergenic, promoter, UTR, etc.) of the reference genome was assessed using bam-stats (from NGSUtilsJ v.0.4.17) [20]. Uniquely mapped reads were used to quantify the gene-level expression employing featureCounts (subread v.2.0.3) [21] with the following parameters: “-s 2 -p –countReadPairs Q 10”. Transcripts per million (TPM) were calculated using length values determined by using the “makeTxDbFromGFF” and “exonsBy” functions in the “GenomicFeatures” library and the “reduce” function in the “GenomicRanges” library in R to find the length of the union of non-overlapping exons for each gene [22].

### 2.6. Data Processing and Analysis

edgeR 4.4.2 was used for the differential expression analysis of the RNA-seq data [23]. Cutoffs were set for FDR < 0.05 and fold change (|log_2_fold-change| > 1) to determine which genes were upregulated or downregulated. Pairwise comparisons were performed between the control group (DMSO), thapsigargin 6 h, and thapsigargin 24 h groups, generating a list of differentially expressed genes (DEGs) for each time point and cell line, which were used to create volcano plots in R using ggplot2 (v3.5.2). For comparisons of DEGs across cell lines, we generated upset plots using the UpSetR (v1.4.0) package [24]. Genes changed uniquely in each cell line were extracted, and these lists were analyzed in R using Gene Ontology and Gene Set Enrichment Analysis (fGSEA (v1.32.4) in R, GO terms string-db [25] and EnrichR [26]). R scripts are numbered in the order they were used in the workflow and are provided on GitHub (https://github.com/kalwatlab/endocrine-ER-stress/, accessed on 26 September 2025).

### 2.7. Interactive Web Tool Design

To generate a web tool to interact with the RNAseq data, we used Shiny (v1.11.1) in R to integrate the data and analyses from our pipeline. The app includes tabs for volcano plot, gene set dot plot generation, data table, pairwise cell line comparisons, interactive upset plots, and GSEA results. The web tool is available at https://diabetes-detectives.shinyapps.io/endocrine-ER-stress/, accessed on 26 September 2025.

### 2.8. Statistical Analysis

Graphed data are expressed as mean ± SD. Data were evaluated using one- or two-way ANOVA as indicated, with appropriate post hoc tests, and considered significant if *p* < 0.05. GraphPad Prism 10 was used to perform statistical tests for qPCR graphs. Statistics for transcriptomics data was performed in R using edgeR with the design set to model.matrix(~0 + group, data = dge_list$samples) and fit with the glmQLFit() function.

## 3. Results

### 3.1. Thapsigargin Induces ER Stress in Multiple Endocrine Cell Lines from Distinct Tissue Types

**Figure 1:**
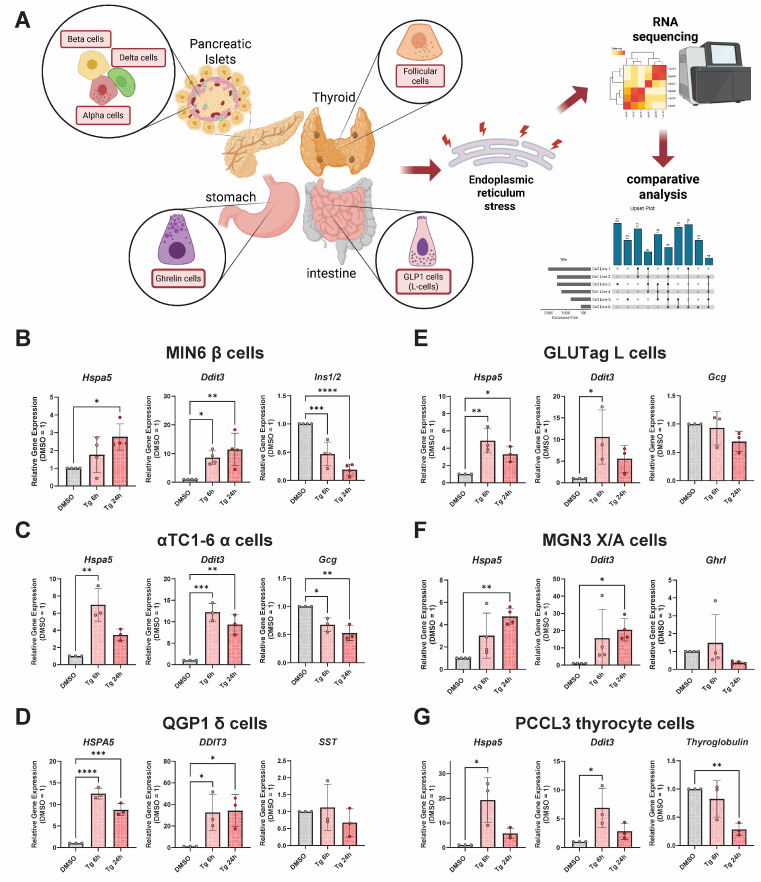
Selection of representative endocrine cell lines and validation of induction of UPR^ER^ genes. (**A**) Cartoon model depicting selection o endocrine cell types and their tissues of origin. Created in BioRender. Kalwat, M. (2025) https://BioRender.com/4p04yyh, accessed on 26 September 2025. (**B**–**G**) qPCR of genes in the lines for validation. Data are the mean ± SD of N = 3–4. *, *p* < 0.05; **, *p* < 0.01; ***, *p* < 0.001; ****, FDR < 0.0001 vs. DMSO by one-way ANOVA with Dunnett’s multiple comparisons test. Data in panels (**D**,**E**) are replotted from Rodrigues-dos-Santos et al. [27], with permission.

To study endocrine cell-type-specific responses to ER stress, we selected cell lines that represent the pancreatic islet (α-cells: αTC1-6; β-cells: MIN6; δ-cells: QGP1), the gut (L-cells: GLUTag; X/A-cells: MGN3-1), and the thyroid (thyrocytes: PCCL3) (Figure 1A). To induce the UPR^ER^ in different endocrine cell types, we used a canonical ER stressor, thapsigargin, which inhibits the SERCA2 Ca^2+^ ATPase, causing ER Ca^2+^ depletion. We confirmed our thapsigargin was active by performing cell viability/death assays in MIN6 and αTC1 cells (Figure S1). We found by RT-qPCR that treatment with thapsigargin for either 6 or 24 h induced canonical UPR^ER^ genes, including *Hspa5* (BiP) and *Ddit3* (CHOP), across all cell lines (Figure 1B–G). Thapsigargin also significantly reduced expression of cell-type-specific hormones in some cell lines, including *Ins1/2* in MIN6 β-cells, *Gcg* in αTC1 α-cells, and thyroglobulin in PCCL3 thyrocytes (Figure 1B,C,G).

**Figure 2:**
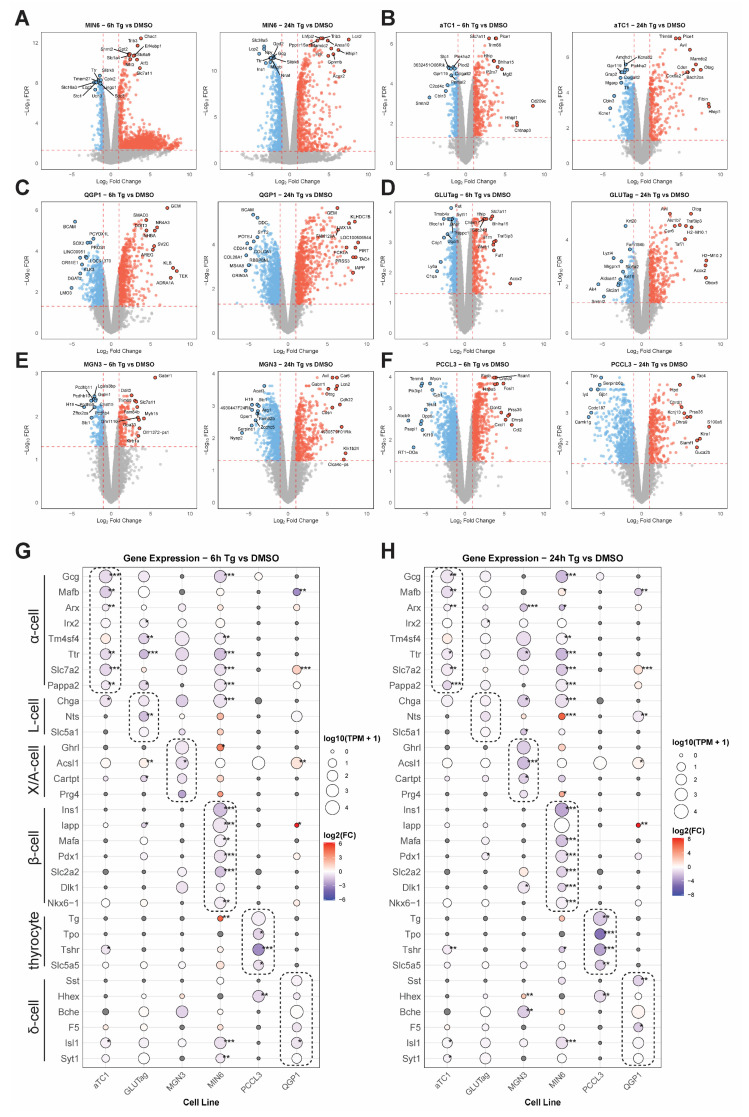
Endocrine cell type responses to thapsigargin-induced ER stress. Volcano plots of differentially expressed genes (DEGs) at 6 and 24 h of thapsigargin (Tg) treatment for (**A**) MIN6 β-cells, (**B**) αTC1 α-cells, (**C**) QGP-1 δ-cells, (**D**) GLUTag L-cells, (**E**) MGN3 X/A-cells, and (**F**) PCCL3 thyrocytes. Cell-type-specific marker gene expression for each cell line is shown for 6 h (**G**) and 24 h (**H**) of Tg treatment. Data are the mean of three independent biological replicates. *, FDR < 0.05; **, FDR < 0.01; ***, and FDR < 0.001 vs. DMSO by edgeR analysis.

Having validated that Tg induces the UPR^ER^ in each of the selected lines, we performed bulk RNA-seq on all samples (DMSO, Tg 6 h, Tg 24 h; N = 3 per cell line). The transcriptomic data were processed and analyzed separately for each cell line using edgeR to identify differentially expressed genes for Tg 6 h vs. DMSO and Tg 24 h vs. DMSO for each cell line (Figure 2A–F). All edgeR results were merged into a supplemental table (Table S3). Table S3 includes a sheet that shows the full list of all genes in all lines, as well as a “summary” sheet that only shows genes that were significantly changed (log_2_FC > 1 and FDR < 0.05) in at least one line. We found that each cell line expressed its expected cell-type-specific markers [28,29,30,31], including *Gcg*, *Mafb*, and *Arx* for α-cells; *Gcg*, *Chga*, and *Nts* for L-cells; *Ghrl*, *Acsl1*, *Cartpt*, and *Prg4* for X/A (ghrelin) cells; *Ins1*, *Mafa*, and *Slc2a2* for β-cells; *Tg* (thyroglobulin), *Tpo*, and *Tshr* for thyrocytes; and *Sst*, *Hhex*, and *Bche* for δ-cells (Figure 2G,H). At both 6 and 24 h exposure to thapsigargin, these cell type markers were the most significantly decreased by Tg in α-cells, β-cells, and thyrocytes, while ghrelin cell, L-cell, and δ-cell markers were less affected (Figure 2G,H).

**Figure 3:**
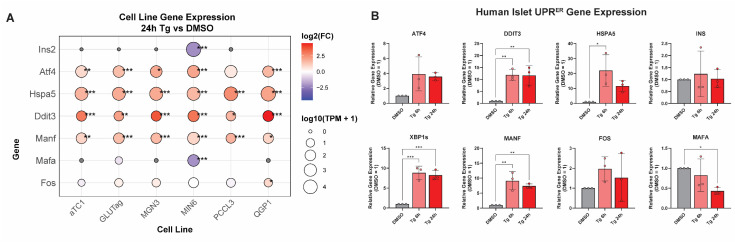
UPR^ER^ and β-cell genes altered across endocrine lines are consistent in human islets treated with thapsigargin. (**A**) Subset of ER stress and β-cell genes for comparison to gene expression in human islets treated with Tg for 6 and 24 h in (**B**). (**B**) Human islets treated with Tg (1 μM) for 6 or 24 h and gene expression quantified by RT-qPCR. *, *p* < 0.05; **, *p* < 0.01; ***, *p* < 0.001 vs. DMSO by one-way ANOVA with Dunnett’s multiple comparisons test.

To confirm that these cell lines exhibit ER stress responses representative of primary cells, we compared selected genes from our transcriptomics to qPCRs of those genes in similarly treated human islets, which are composed mainly of β-, α-, and δ-cells. We assessed this by comparing the expression of ER stress genes at the 6 and 24 h time points in our cell lines (Figure 3A) to that of human islets treated similarly (Figure 3B). In agreement with previous findings [32,33], Tg induced the UPR^ER^ in primary human islets. Following these validations, we next used multiple approaches to examine the similarities and differences in responses to Tg across the different endocrine lines.

### 3.2. Integrating Cell-Type-Specific UPR^ER^ Transcriptomic Responses Identifies Common and Unique Gene Signatures

**Figure 4:**
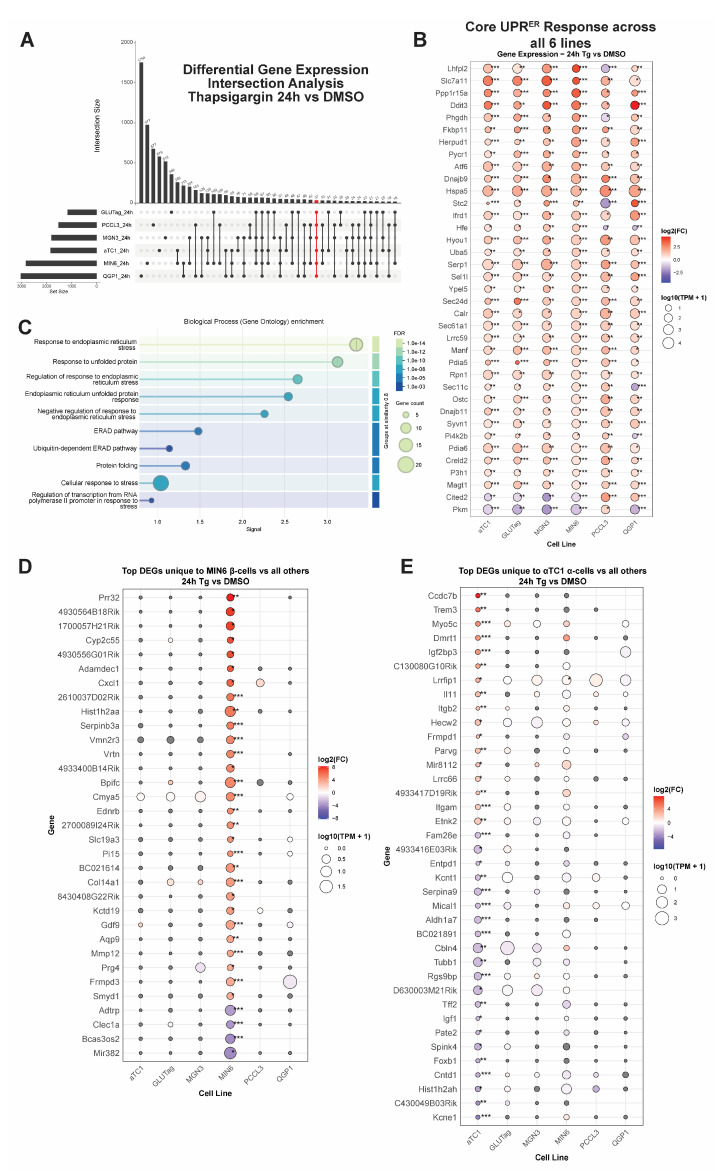
(**A**) Upset plot analysis comparing significantly differentially expressed genes (DEGs) after 24 h of Tg treatment across all 6 cell types. Highlighted in red is the set of DEGs that is common to all cell lines. (**B**) Dot plot of DEGs that are shared among all lines. (**C**) Gene ontology term enrichment from String-DB of the gene set shown in (**B**). (**D**) Subset of the top DEGs that are uniquely changed in MIN6 cells at Tg 24 h. (**E**) Subset of the top DEGs that are uniquely changed in αTC1 cells at Tg 24 h. In all dot plots, dot size is proportional to average expression level in transcripts per million (TPMs), and dots are colored by log_2_FC. *, FDR < 0.05; **, FDR < 0.01; ***, and FDR < 0.001 vs. DMSO by edgeR analysis.

To find distinct and shared gene sets, we used an upset plot analysis to identify the number of DEGs unique to each cell line, as well as those in common between all comparisons of cell lines (Figure 4A, Table S4). Genes that are regulated by Tg in common across all tested cell lines included known factors like *Ddit3* (encoding CHOP), *Ppp1r15a*, and *Slc7a11* (Figure 4B). As expected, this common gene set was enriched for ontology terms related to ER protein processing and response to stress (Figure 4C). We also extracted cell-line-unique DEGs (Table S5). A subset of the top up- and downregulated unique genes from the Tg 24 h time point for β-cells and α-cells is shown (Figure 4D,E). For the MIN6 β-cell-unique genes, the molecular function GO terms included tRNA synthetase activity, RNA modifications like N6-methyladenosine, and solute/ion transport (Figure S3A, Table S6). In contrast, for αTC1-cell-unique genes, the molecular function GO terms included carboxypeptidase activity and aldehyde dehydrogenase activity (Figure S3B, Table S6).

### 3.3. Cell-Type Comparisons Identify Concordant and Discordant Responses to UPR^ER^ in β-Cells Versus α-Cells

**Figure 5:**
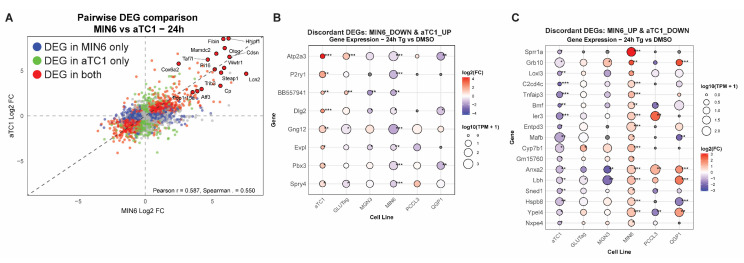
(**A**) Pairwise comparison of log_2_FC of all genes changed by Tg 24 h in MIN6 β-cells versus αTC1 α-cells. Differentially expressed genes (DEGs) in MIN6 only are in blue, αTC1 only are in green, and DEGs in both are in red. (**B**) Discordant DEGs that are downregulated in MIN6 but upregulated in αTC1 after 24 h of Tg treatment. (**C**) Discordant DEGs that are upregulated in MIN6 but downregulated in αTC1 after 24 h of Tg treatment. *, FDR < 0.05; **, FDR < 0.01; ***, FDR < 0.001 vs. DMSO by edgeR analysis.

To identify concordant and discordant gene expressions, we used pairwise log_2_FC comparisons. We correlated the pairwise log_2_FC of DEGs for all lines (Figure S4). The analyses are browsable and exportable via our web app. As an example, we focus on the comparison between MIN6 β-cells and αTC1-6 α-cells (Figure 5A and Figure S5A, Table S7). We found that while many genes altered by thapsigargin were concordant between these two cell types, there was a subset of discordant genes (Figure 5B,C). One of the most discordant genes that was upregulated in α-cells but downregulated in β-cells was *Atp2a3*, which encodes sarcoplasmic/endoplasmic reticulum Ca^2+^ ATPase 3 (SERCA3) (Figure 5B). The SERCA proteins are the main target of thapsigargin, which can inhibit all SERCA isoforms [34]. Interestingly, among all endocrine cells tested, αTC1 and GLUTag cells were the only lines that significantly induced SERCA3. On the other hand, one of the most discordant genes upregulated in β-cells but downregulated in α-cells was *Sprr1a* (Figure 5C). Additionally, when considering the 6 and 24 h time points together, several discordantly upregulated genes in β-cells had to do with cargo receptor activity, including *Cubn*, *Dmbt1*, *Tmprss3*, *Megf10*, and *Loxl3* (Figure 5C and Figure S5C), suggesting β-cells alter their intracellular cargo handling or trafficking substantially differently than α-cells.

**Figure 6:**
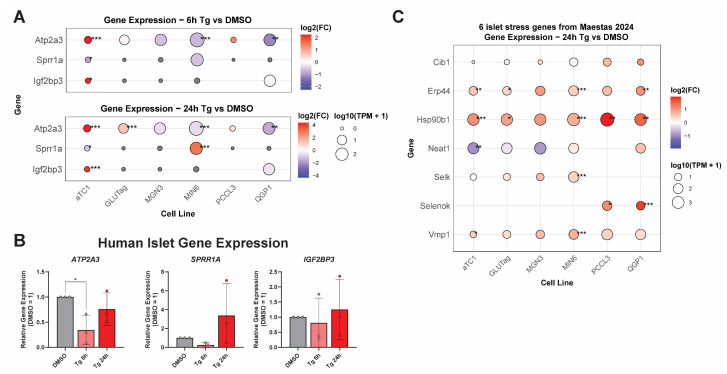
Expression of selected stress response genes in thapsigargin-treated human islets. (**A**) Replotting of Atp2a3, Sprr1a, and Igf2bp3 expression at 6 and 24 h of Tg treatment across all cell lines. (**B**) RT-qPCR in the same human islet samples from Figure 3B to measure the expression of ATP2A3, SPRR1A, and IGF2BP3. Data represent the mean ± SD from three independent donor islet batches. (**C**) Maestas et al. previously identified six core stress genes that were upregulated in human islet α-, β-, and δ-cells. The plot shows the expression of these genes at Tg 24 h for each of our cell lines. Selk is also known as Selenok, depending on the database. Both names are shown to capture the expression data. *, FDR < 0.05; **, FDR < 0.01; ***, FDR < 0.001 vs. DMSO by edgeR analysis.

To determine if changes observed in endocrine cell lines can also be observed in a primary cell system, we measured gene expression in human islets treated with thapsigargin. We selected *ATP2A3*, *SPRR1A*, and *IGF2BP3*, as these genes were altered in specific cell types shared with human islets (α- and β-cells). In αTC1 cells, *Atp2a3* and *Igf2bp3* were each increased at both 6 and 24 h of Tg treatment (Figure 6A). In MIN6 cells, *Sprr1a* was not changed significantly at 6h, but was increased at 24 h of Tg treatment (Figure 6A). In human islets, *ATP2A3* was decreased at 6h of Tg, in greater agreement with MIN6 cell data than αTC1 data (Figure 6B). *SPRR1A* and *IGF2BP3* were not significantly changed in human islets, possibly owing to human donor variability. However, *SPRR1A* trended down at 6 h and up at 24 h, a pattern that appears to agree with MIN6 cell expression data.

We also compared our β-cell and α-cell data with a recent single-cell RNAseq dataset from Maestas et al., where human islets were treated with different stressors, including Tg, bafilomycin A, and cytokines, for 48 h [35]. The authors identified six genes that were commonly upregulated in α-, β-, and δ-cells: *CIB1*, *ERP44*, *HSP90B1*, *NEAT1*, *SELK*, and *VMP1*. We see general agreement in our dataset with these genes being upregulated in αTC1, MIN6, and QGP-1 (Figure S5C). One exception is the lncRNA NEAT1, which was downregulated in αTC1.

### 3.4. Generation of an Interactive Transcriptomics Browser for Endocrine UPR^ER^

**Figure 7:**
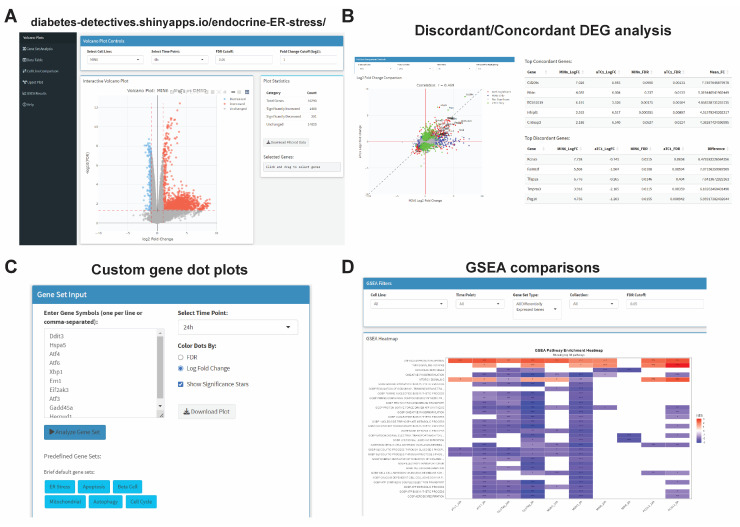
Interactive transcriptomics explorer for endocrine ER stress data. (**A**) The data explorer has multiple interactive plot types (e.g., volcano, pairwise log_2_FC, upset plots). (**B**) Pairwise comparisons allow for export of plots and concordant/discordant genes for selected cell line and time points. (**C**) Custom gene set analysis that generates dot plots. Genes are kept in the order given, and dot size is log_10_ (TPMs + 1) and colored by log_2_FC. (**D**) Filterable GSEA results for each time point and cell type with export capabilities. *, FDR < 0.05; **, FDR < 0.01; ***, FDR < 0.001 vs. DMSO by edgeR analysis.

To provide a resource to the UPR^ER^ field, we integrated our RNAseq data into an interactive web tool using Shiny [36], accessible at https://diabetes-detectives.shinyapps.io/endocrine-ER-stress/, accessed on 26 September 2025. This tool has functions for browsing volcano plots (Figure 7A), pairwise comparisons of log_2_FC data between cell lines (Figure 7B), a custom gene set dot plot generation tool (Figure 7C), GSEA results across cell lines (Figure 7D), a filterable data table viewer for all cell line edgeR results and TPMs, and an interactive upset plot viewer.

## 4. Discussion

The UPR^ER^ is engaged in T1D and T2D in the β-cell and is an attractive source of pharmacological targets for diseases, including diabetes [3,6,37]. Compared to related cell types, β-cells have a unique reliance upon the UPR^ER^ as they handle the daily task of insulin production [38]. The differences and similarities between β-cells and other specialized secretory cells are in part due to their requirement for exquisite balance in this pathway. Heterogeneity of genetic and environmental backgrounds likely influences the resistance or susceptibility of β-cells to stress. For example, β-cells require XBP1 to maintain identity [39]; however, IRE1α deletion can help protect β-cells in T1D models [40]. The β-cell UPR^ER^ is also engaged in monogenic forms of diabetes, such as mutant insulin-induced diabetes of youth (MIDY) [41], where mutations in the INS gene cause misfolding of the insulin protein, leading to activation of UPR^ER^ and β-cell dysfunction [42]. Therefore, investigations into the effects of pharmacological or genetic modulation of the UPR^ER^ in β-cells have broad mechanistic and therapeutic implications.

Pancreatic islet β-cells and α-cells are well known to differ in their responses to stress. For example, in T1D, the immune system recognizes β-cells and destroys them but does not target α-cells [43]. The contributors to this phenomenon have been speculated to include higher expression of anti-apoptotic, viral recognition, and innate immune response genes in α-cells, as well as a relatively higher response to ER stress in β-cells [8]. In our study, we identified candidate genes that may contribute to α-cell-specific vs. β-cell-specific responses to canonical ER stress. For example, we found that *Atp2a3* (SERCA3) is induced in α-cells but downregulated in β-cells (Figure 5B and Figure 6A,B). SERCA3 was previously shown to be expressed in mouse pancreatic islet β-cells but absent in islet α-cells [44], although human islet scRNAseq suggests that α-cells and δ-cells express the *ATP2A3* gene [30,45]. Perhaps α-cells’ upregulation of SERCA3 could contribute to their known resistance to ER stress [8]. Indeed, small molecule SERCA activators are under investigation as potential β-cell therapeutics in diabetes [46,47]. Another gene selectively upregulated in α-cells was *Igf2bp3* (Figure 4E). Igf2bp3 plays a role in binding to N6-methyladenosine (m6A)-modified mRNA [48]. m6A modifications have become of increasing interest in T1D and T2D biology due to their potential regulatory role in pancreatic β-cells [49,50]. Although we did not observe a significant induction of *IGF2BP3* in Tg-treated human islets, it is important to consider that multiple cell types in the islet could obscure expression changes, and/or human donor variability could reduce the ability to detect changes. The role(s) for m6A regulation in α-cells and how *Igf2bp3* may be important during islet stress are unknown.

One of the most discordant genes upregulated in the β-cell but downregulated in α-cells was *Sprr1a,* which encodes small proline-rich protein 1A. SPRR1A is involved in keratinocyte function as a structural protein [51], although new functions have recently been identified. In mice with autophagy-deficient β-cells (inducible knockout of *Atg7* only in β-cells), *Sprr1a* was found to be one of the most highly upregulated genes after 2-6 weeks of induced knockout [52]. The authors confirmed this at the protein level, where Sprr1a was upregulated in db/db mouse islets and in wild-type mice subjected to chemically induced insulin resistance. Additionally, we observed that *SPRR1A* expression appeared to increase in two of three Tg-treated human donor islets. Taken together, these findings suggest that *Sprr1a* is a stress-responsive gene, possibly specific to β-cells. None of the other endocrine cell types we tested had increased *Sprr1a* expression.

## 5. Conclusions

In conclusion, comparisons of cell-type-specific transcriptomics downstream of the UPR^ER^ can uncover potentially new regulatory mechanisms driving secretory cell survival or dysfunction. To our knowledge, this study is the first comparative transcriptomics analysis of multiple distinct endocrine cell type responses in an ER stress model at multiple time points. This approach may lead to new gene targets for disease treatments or biomarkers of stressed cells. These data represent a useful resource to support multiple fields, including general secretory cell biology, neuroendocrine tumors (NETs), and metabolic and endocrine diseases.

Limitations of the study: Our raw data is from cell lines, although we compared the data to primary human islet gene expression data. Induction of ER stress in our study depended on a pharmacological agent, thapsigargin. While this compound has been used for decades for this purpose, it may not fully replicate the UPR^ER^ that occurs normally in vivo. Additionally, inferences from this data are also dependent on RNA expression, which does not always reflect protein expression. Future work will require validation studies for these signature genes at the protein level in specific endocrine cell types from primary tissues and using additional ER stress model systems.

## Data Availability

RNA-seq data associated with this manuscript are publicly available under NCBI GEO identifiers GSE238017 and GSE194200. The code is provided on GitHub (https://github.com/kalwatlab/endocrine-ER-stress/, accessed on 26 September 2025). All other supplementary materials are provided as part of this manuscript or its online repository. Claude.ai was used to optimize R scripts. The authors reviewed and edited the scripts, as needed, and take full responsibility for the content of the publication.

## Supplementary Materials

The following supporting information can be downloaded at: https://www.mdpi.com/article/10.3390/cells14191529/s1, Figure S1—related to Figure 1. Figure S2—related to Figure 4. Figure S3—related to Figure 4. Figure S4—related to Figure 5. Figure S5—related to Figure 5. Table S1. Human Islet Checklist; Table S2. Primers; Table S3. All cell line edgeR results and TPMs; Table S4. Upset 6 h 24 h all lines intersection; Table S5. Unique genes all lines; Table S6. MF GO terms MIN6 and αTC1; Table S7. MIN6 vs. αTC1 concordant and discordant genes.

## Abbreviations

The following abbreviations are used in this manuscript:

UPR^ER^	Unfolded protein response of the endoplasmic reticulum
T1D	Type 1 diabetes
T2D	Type 2 diabetes
GSEA	Gene set enrichment analysis
GLP1	Glucagon-like peptide 1
Tg	Thapsigargin
