# Supplementary material for: Unique and Conserved Endoplasmic Reticulum Stress Responses in Neuroendocrine Cells
Source: Cells. 2025 Sep 30;14(19):1529. doi: 10.3390/cells14191529 (PMC12524277; doi:10.3390/cells14191529)
Supplement: Supplementary file 1 [file cells-14-01529-s001.zip › Rodrigues dos Santos Endocrine ER stress transcriptomics SUPP INFO R1.pdf]

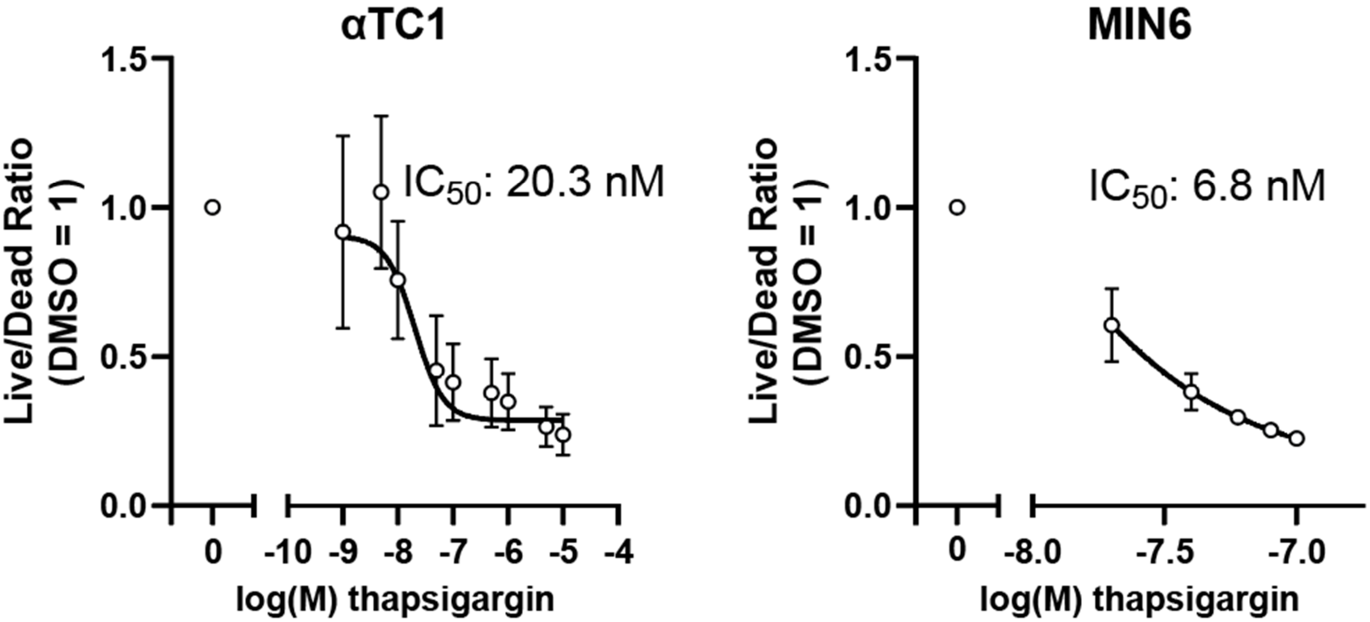

Figure S1 – Related to Figure 1. αTC1 and MIN6 cells were exposed to concentration response curves of thapsigargin for 24 h. Cells were analyzed with the MultiTox-Fluor assay which measures viable and dead cells simultaneously, enabling a Live/Dead ratio to be calculated. Data represent the mean ± SD of two independent assays.

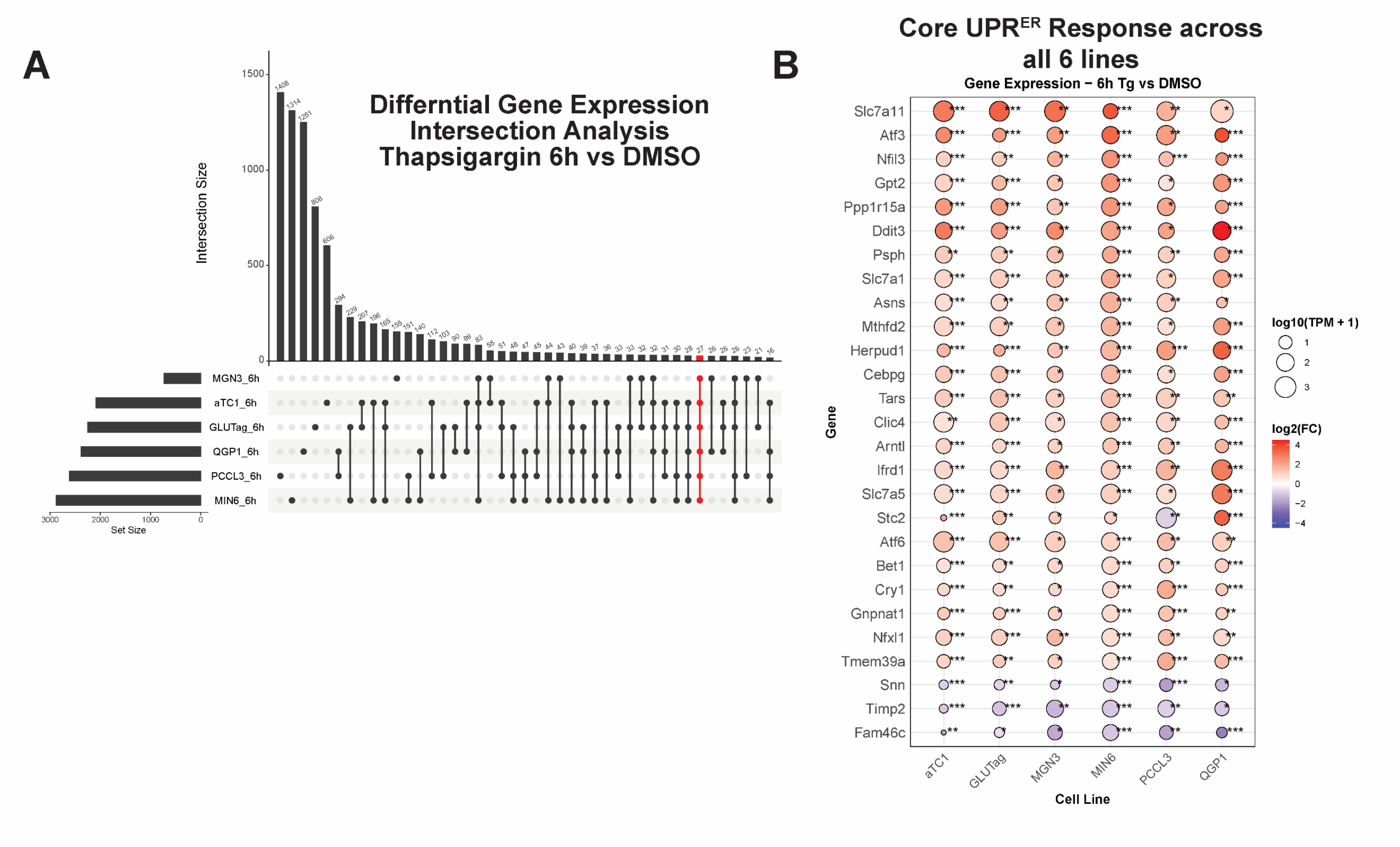

Figure S2 – Related to Figure 4. A) Upset plot of DEGs in all cell lines at Tg 6h. Highlighted in red is the set of DEGs that is common to all cell lines. **B)** Dot plot of DEGs shared between all 6 cell lines at Tg 6h. FDR<0.05; **, FDR<0.01; ***,FDR<0.001 vs DMSO by edgeR analysis.

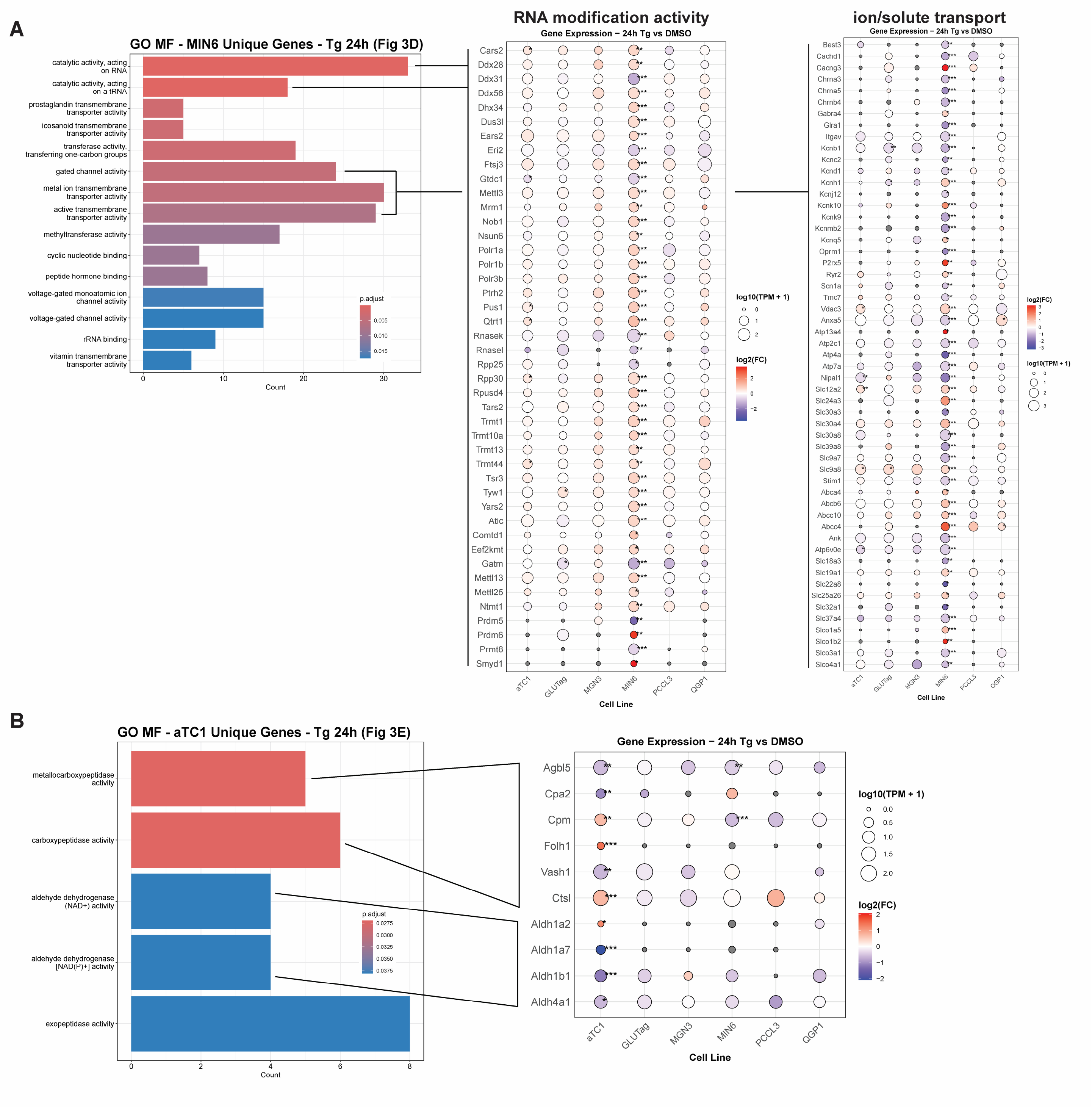

Figure S3 – Related to Figure 4. A) Molecular Function (MF) Gene Ontology (GO) enrichment for entire list of MIN6 unique DEGs (related to Fig 3D) at Tg 24 h. Dot plots show gene expression for genes within selected terms for RNA modifying activity and ion/solute transport activity. **B)** Similar to (**A**), MF GO enrichment terms for αTC1 unique DEGs at Tg 24 h. Dot plots show expression for genes comprising the carboxypeptidase activity terms and aldehyde dehydrogenase activity terms. FDR<0.05; **, FDR<0.01; ***,FDR<0.001 vs DMSO by edgeR analysis.

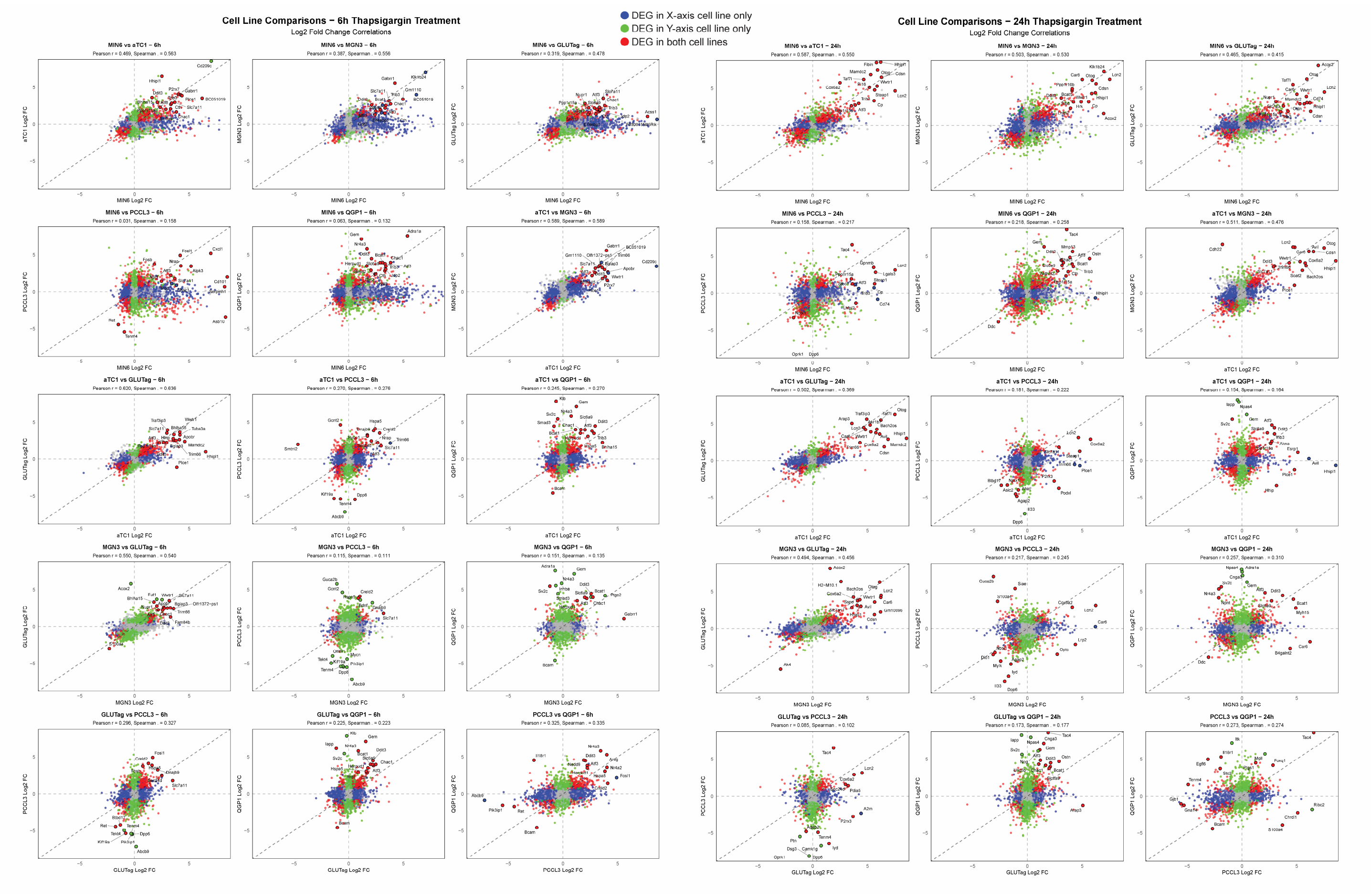

Figure S4 – related to Figure 5. Pairwise comparison of log2FC gene expression data for every combination of the 6 tested cell lines. Tg 6h data shown on the left and Tg 24 h data shown on the right. Differentially expressed genes (DEGs) in the X-axis cell line only are in blue, the Y-axis cell line only are in green, and red color is used for genes that are DEGs in both lines.

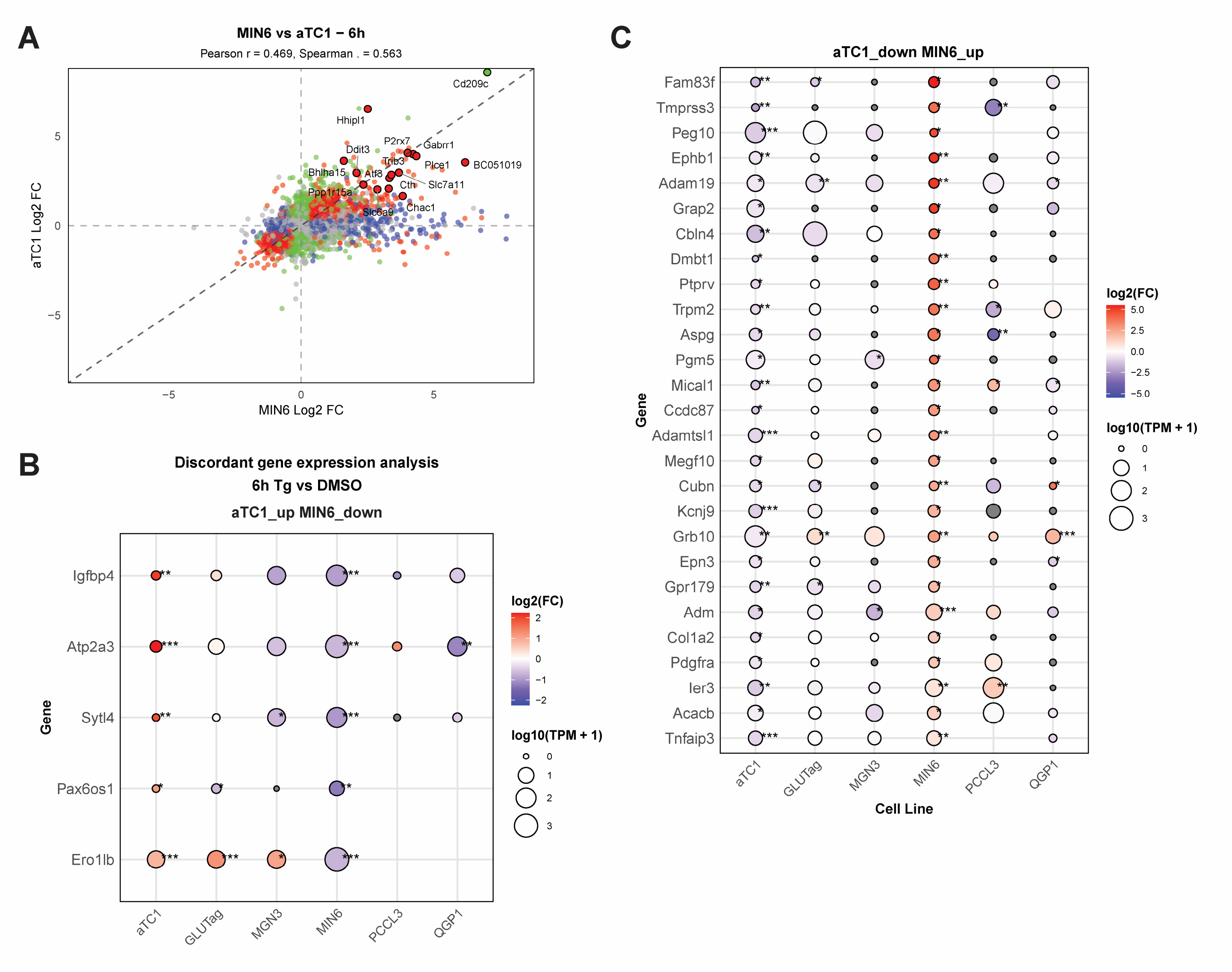

Figure S5 – related to Figure 5. A) Larger plot for pairwise comparison of MIN6 and αTC1 cells treated with Tg for 6 h, as seen in Fig S4. **B)** Discordant DEGs that are downregulated in MIN6 but upregulated in αTC1 after 6 h of Tg treatment. **C)** Discordant DEGs that are upregulated in MIN6 but downregulated in αTC1 after 6 h of Tg treatment.
