# Supplementary material for: Unique and Conserved Endoplasmic Reticulum Stress Responses in Neuroendocrine Cells
Source: Cells. 2025 Sep 30;14(19):1529. doi: 10.3390/cells14191529 (PMC12524277; doi:10.3390/cells14191529)
Supplement: Supplementary file 1 [file cells-14-01529-s001.zip › Table S1. Human Islet checklist.pdf]

humanChecklist for reporting human islet preparations used in research

Adapted from Hart NJ, Powers AC (2018) Progress, challenges, and suggestions for using human islets to understand islet biology and human diabetes. Diabetologia https://doi.org/10.1007/s00125-018-4772-2

Islet preparation	1	2	3	4	5	6	7	8[a]
**MANDATORY INFORMATION**								
Unique identifier	HP-23153-01	HP-23166-01	SAMN34033792					
Donor age (years)	51	58	39					
Donor sex (M/F)	Female	Female	Male					
Donor BMI (kg/m^2)	26.4	28.8	33.4					
Donor HbA_{1c} or other measure of blood glucose control	5.3%	5.6%	5.0%					
Origin/source of islets[b]								
Islet isolation centre	Prodo Labs	Prodo Labs	Southern California Islet Cell Resource Center					
Donor history of diabetes? Please select yes/no from drop down list	No	No	No					
**If Yes, complete the next two lines if this information is available**								
Diabetes duration (years)								
Glucose-lowering therapy at time of death[c]								
**RECOMMENDED INFORMATION**								
Donor cause of death	stroke	stroke	head trauma					

Warm ischaemia time (h)								
Cold ischaemia time (h)			12h 41m					
Estimated purity (%)	85	80-85	80					
Estimated viability (%)	95	95	97					
Total culture time (h)[d]								
Glucose-stimulated insulin secretion (static culture by IIDP)[e]			2.2					
Handpicked to purity? Please select yes/no from drop down list	Yes	Yes	Yes					
Additional notes:<br>Related to Figure:								
Additional notes<br>-Culture time prior to shipment			2d 7h					

[a]If you have used more than eight islet preparations, please complete additional forms as necessary
[b]For example, IIDP, ECIT, Alberta IsletCore
[c]Please specify the therapy/therapies
[d]Time of islet culture at the isolation centre, during shipment and at the receiving laboratory
[e]Please specify the test and the results
